# Supplementary material for: Polyclonal epitope mapping reveals temporal dynamics and diversity of human antibody responses to H5N1 vaccination
Source: Cell Rep. Author manuscript; Available in PMC 2021 Feb 17. (PMC7888560; doi:10.1016/j.celrep.2020.108682)
Supplement: 1 [file NIHMS1667159-supplement-1.pdf]

Cell Reports, Volume *34*

Supplemental Information

Polyclonal epitope mapping reveals temporal dynamics and diversity of human antibody responses to H5N1 vaccination

Julianna Han, Aaron J. Schmitz, Sara T. Richey, Ya-Nan Dai, Hannah L. Turner, Bassem M. Mohammed, Daved H. Fremont, Ali H. Ellebedy, and Andrew B. Ward

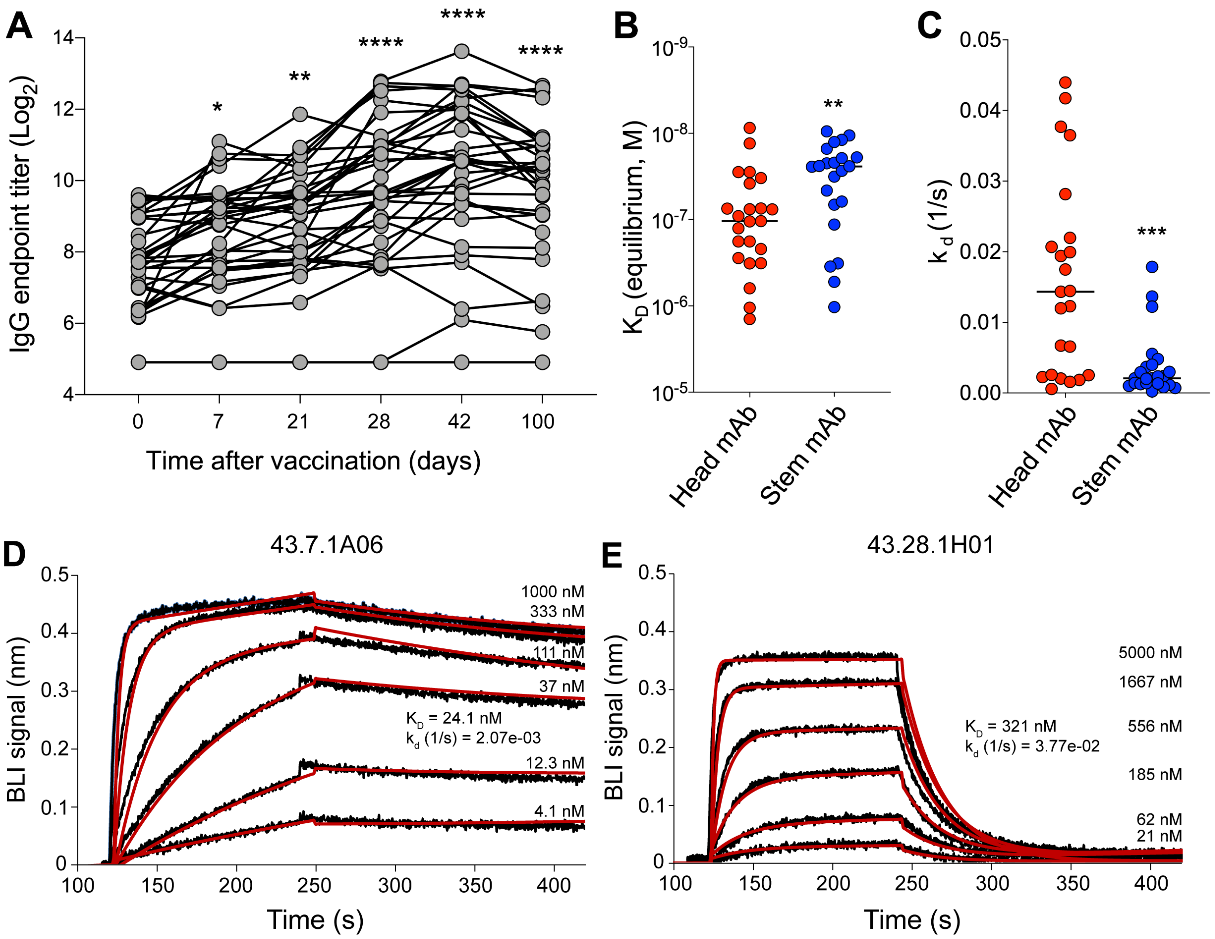

Supplemental Figure 1. Related to Figure 1. Affinity assessment of mAbs from subjects 4, 28, 36, and 43.

(A) ELISA binding titers of serum IgG to recombinant H5 HA over the course of vaccination.

(B-C) affinity measurements (B) and off-rates (C) of H5 HA stem- (blue) and head-specific (red) plasmablast-derived monoclonal Fab fragments isolated at days 7 and 28 binding to recombinant H5 HA. (D-E) Representative biolayer interferometry (BLI) binding curves of monoclonal Fab fragments 1A06 (D) and 1H01 (E) isolated from subject 43 at days 7 and 28 binding to recombinant H5 HA. p-values were determined by unpaired t tests: * p-value <0.05, ** p-value < 0.01, *** p-value < 0.001, **** p-value < 0.0001, N.S. non-significant.

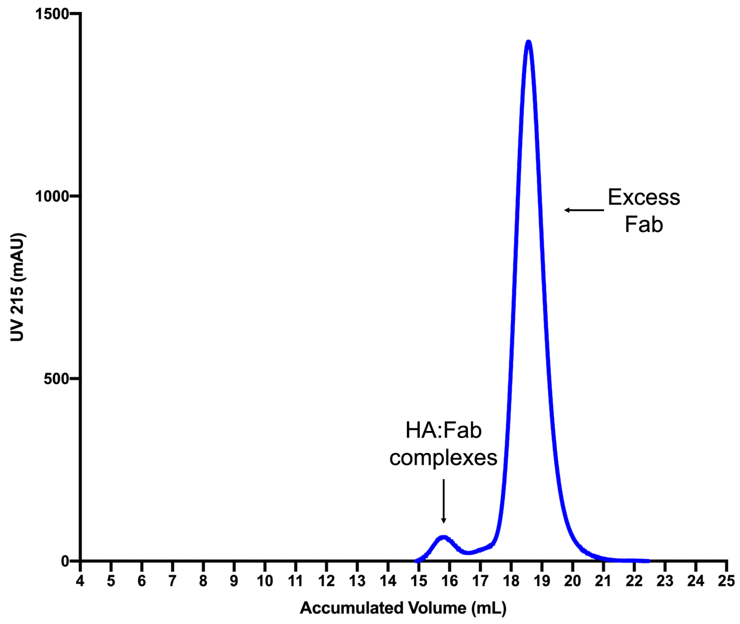

Supplemental Figure 2. Related to Figure 2. Purification of polyclonal immune complexes. (A) Example chromatogram from size exclusion chromatography separation of polyclonal immune complexes. Peaks corresponding to elution of immune complexes and excess Fab are labeled.

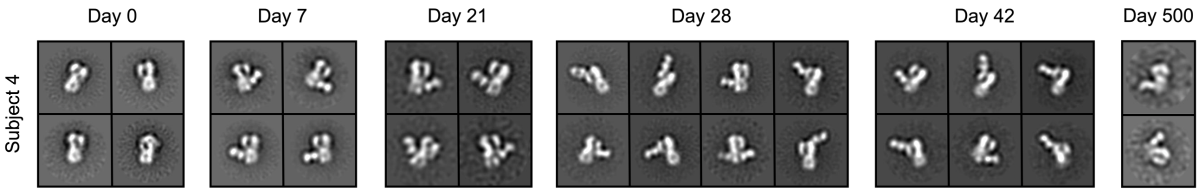

Supplemental Figure 3. Related to Figure 3. Subject 4 2D class averages over the course of H5N1 vaccination.
(A) Example 2D class averages of immune complexes or unbound H5 HA at corresponding time points after H5N1 vaccination in subject 4.

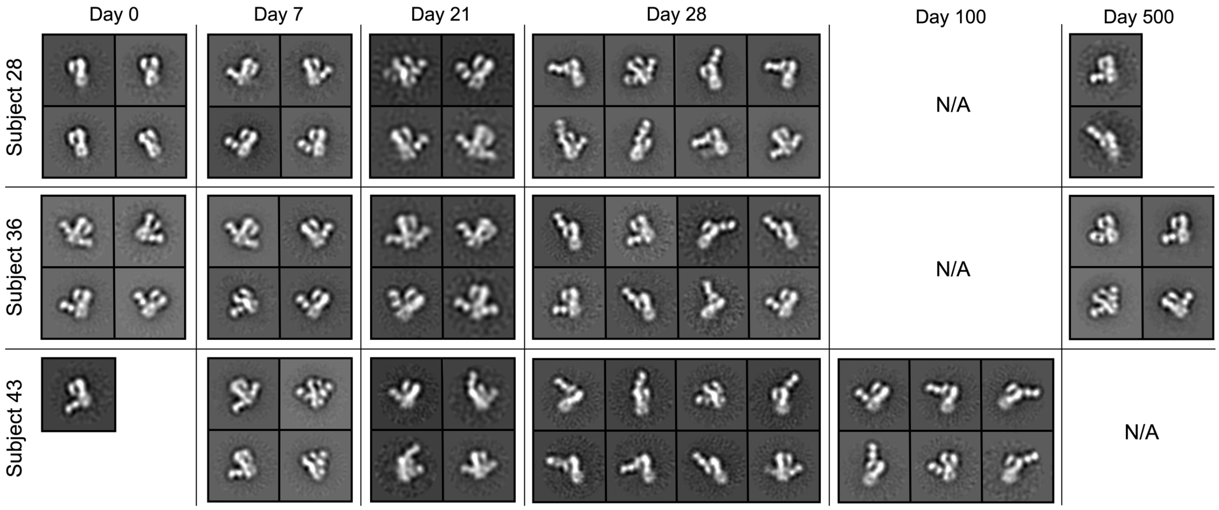

Supplemental Figure 4. Related to Figure 4. Subjects 28, 36, and 43 2D class averages over the course of H5N1 vaccination.
(A) Example 2D class averages of immune complexes or unbound H5 HA at corresponding time points after H5N1 vaccination in subjects 28, 36, and 43.

A

mAb	Variable Gene Usage	Epitope
Subject 4 day 7 1C01	IGHV1-69 IGKV1-5	V_H 1-69 stem
Subject 4 day 28 1F03	IGHV3-33 IGLV2-14	RBS
Subject 4 day 28 1H09	IGHV3-33 IGLV1-40	RBS
Subject 43 day 7 1A06	IGHV1-69 IGLV2-14	V_H 1-69 stem
Subject 43 day 7 1B02	IGHV1-69 IGKV3-20	V_H 1-69 stem
Subject 43 day 7 1B04	IGHV1-69 IGKV1-39	V_H 1-69 stem
Subject 43 day 28 1B02	IGHV4-59 IGKV1-33	Vestigial Esterase
Subject 43 day 28 1A08	IGHV3-33 IGLV1-47	RBS
Subject 43 day 28 1A02	IGHV1-18 IGKV3-15	RBS
Subject 43 day 28 1E01	IGHV3-43 IGKV1-5	RBS
Subject 43 day 28 1H01	IGHV3-20 IGKV1-5	RBS

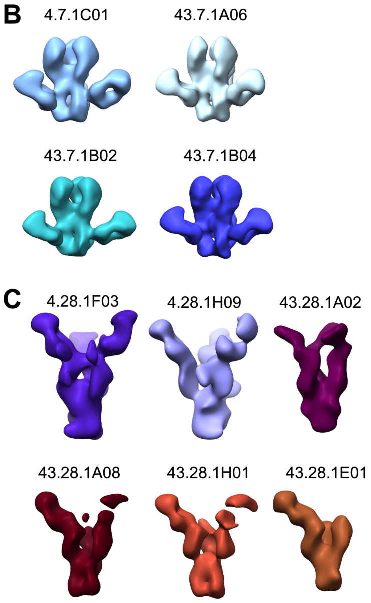

Supplemental Figure 5. Related to Figure 5. Subject 43 mAbs target discrete regions on HA.

(A) Table of mAbs isolated from plasmablasts of subjects 4 and 43. Variable gene usages and EM-mapped epitopes are listed. (B-C) 3D reconstructions of immune complexes from day 7 (B) or day 28 (C) plasmablasts with recombinant H5 HA (A/Indonesia/5/2005).

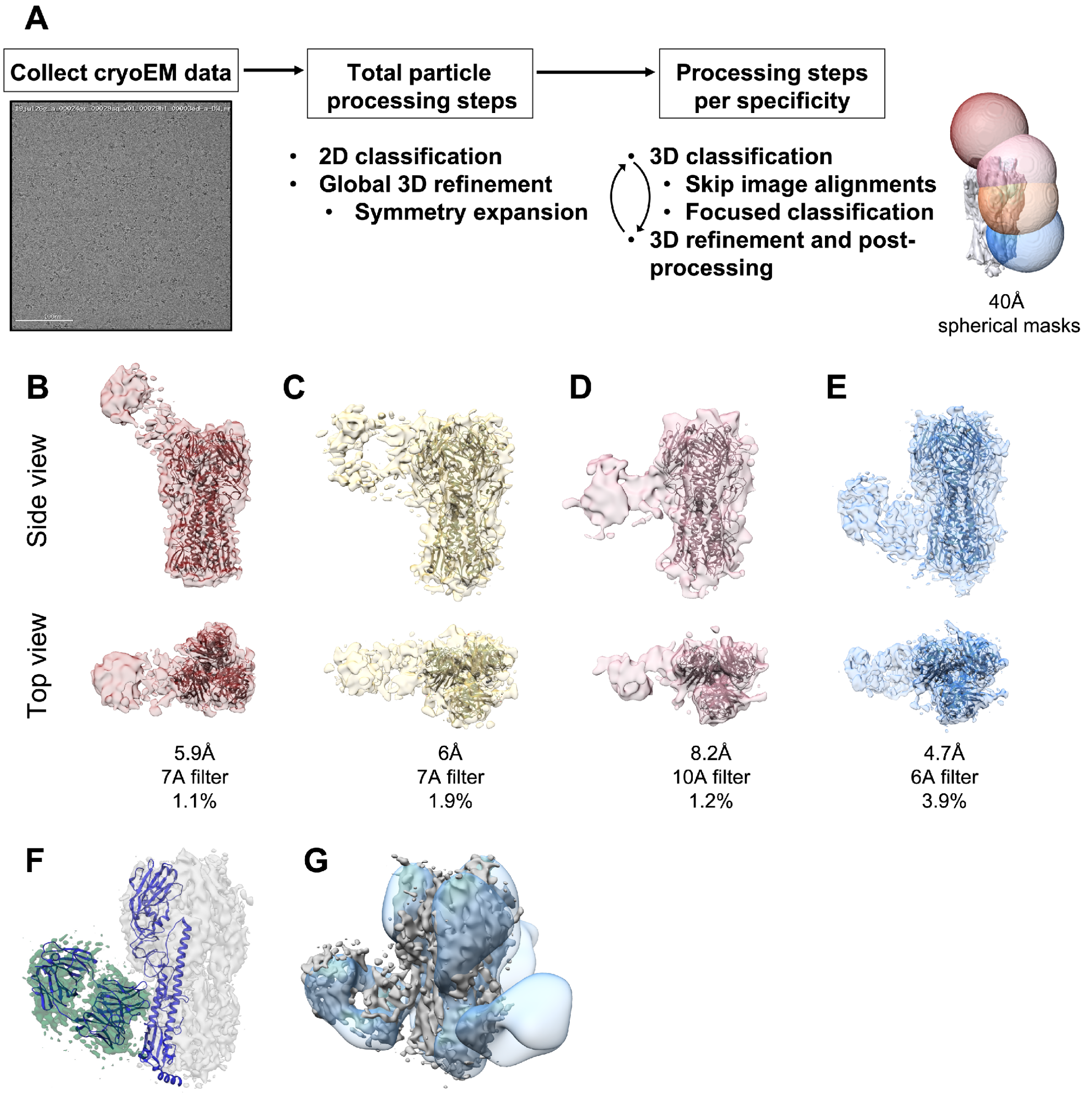

Supplemental Figure 6. Related to Figure 6. CryoEM maps of polyclonal immune complexes.

(A) Workflow of cryoEM focused classification and refinement: Briefly, after cryoEM data collection and initial processing steps, particles are classified and cleaned in 2D. All particles are masked around the HA trimer, aligned to a reference HA trimer (PDB 4K62), and C3 symmetry expanded. For focused classification, particles are classified in 3D without global image alignment using a 40 Å spherical mask around regions of anticipated pAbs. For focused refinement, 3D classes of individual immune complexes are further refined with a mask around the immune complex. Final maps of specific immune complexes are then produced from multiple iterations of focused classification and refinement. Scale bar on micrograph denotes 100nm. (B-E) CryoEM maps of pAbs in complex with recombinant H5 HA (A/Indonesia/5/2005). Maps were low-pass filtered to 9A for the RBS-specific (B), 8A for the lateral patch-specific (C), 10A for the vestigial esterase-specific (D), and 6A for the stem-specific (E) immune complexes. (F) CryoEM map of

stem-specific immune complex in gray and green with CR9114 ribbon diagram docked into EM density (PDB 4FQI). (G) CryoEM map of stem-specific immune complex in grey compared with subject 4 day 7 mAb 1C01 complexed with H5 HA in blue.

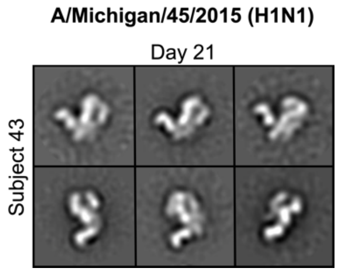

Supplemental Figure 7. Related to Figure 7. Subject 43 2D class averages of immune complexes with heterosubtypic H1 HA.
(A) Example 2D class averages of H1 HA (A/Michigan/45/2015) immune complexes at day 21 in subject 43.
